# Family members’ experiences of courtesy stigma associated with mental illness

**DOI:** 10.4102/hsag.v28i0.2184

**Published:** 2023-08-29

**Authors:** Wada Gaolaolwe, Eva Manyedi, Maserapelo Serapelwane

**Affiliations:** 1 Department of Psychiatric Mental Health Nursing, Lobatse Institute of Health Sciences, Lobatse, Botswana; 2 Department of Nursing, Faculty of Health Sciences, North-West University, Mafikeng, South Africa

**Keywords:** courtesy stigma, mental illness, family, labelling, self-stigma, mental illness discrimination, stigma by association, mental illness stereotypes

## Abstract

**Background:**

The stigma of mental illness has been in existence from medieval times to date and it is extended to families of people diagnosed with mental illness. Families with a member diagnosed with a mental illness experience courtesy stigma of mental illness and it affects the quality of their lives.

**Aim:**

This study aimed to explore and describe the experiences of courtesy stigma of families with a member diagnosed with a mental illness in Lobatse, Botswana.

**Setting:**

The study was conducted at a psychiatric hospital in Lobatse, Botswana.

**Methods:**

A qualitative contextual phenomenological design was used for this study. The population comprised of members from families with a person diagnosed with a mental illness and the sample size was 15 participants. Semi-structured in-depth individual interviews were conducted telephonically.

**Results:**

The study yielded three main themes and related subthemes. The themes were: families’ experiences of received stigma, families’ experiences of stigma by association, and families’ experiences of internal stigma.

**Conclusion:**

Families with a member diagnosed with mental illness experience received stigma, associated stigma and internal stigma. The families experienced that they received dehumanising labels from the public because of their association with their mentally ill family members.

**Contribution:**

With the insights gained from the findings of this study, programmes can be developed that raise awareness on stigma of mental illness and to promote support of families of people diagnosed with a mental illness.

## Introduction

Stigma is a social phenomenon in which the individuals who are viewed by the public to possess attributes that are perceived as inferior are treated negatively and unequally (Stutterheim & Ratcliffe 2021). Historically, mental illness stigma has always existed and pervaded literature from medieval times to date (Yin, Li & Zhou 2020). Generally, stigma of mental illness has existed for centuries although the concept ‘stigma’ was first articulated by Erving Goffman around the 1960s (Yin et al. 2020). Even though the conceptualisation of stigma is reported to have been coined around the 1960s, there is evidence from literature that the term was originated by the ancient Greeks to refer to bodily branding or mark meant to expose the bearer that they were ritually polluted, were criminals or had to be avoided (Goffman 1963). Later on in the Christian era, Goffman (1963) reported that the term referred to eruptive skin blossoms or physical infirmity, which made the affected to look different from others, hence faced stigmatisation. The concept of stigma continues to be redefined owing to evolving moral concepts, culture and dynamic life changes (Becker et al. 2019; Goffman 1963). Today, the term is as articulated by Goffman and still carries the same original meaning, although it now denotes the disgrace that the stigmatised individuals or their families suffer as opposed to the body branding evidence. The negative attributing stereotypes, labelling and discriminations are not only directed towards people with mental illness but is often extended to their family members by their association with them (Stutterheim & Ratcliffe 2021; Van Der Sanden et al. 2015).

Literature refers to the stigma towards families with a member diagnosed with mental illness as the stigma by association, family stigma or courtesy stigma (Yin et al. 2020). Courtesy stigma results in shame of family members and their avoidance of social events (Yin et al. 2020). Generally, families experiencing courtesy or family stigma suffer the impact of both public and self-stigma, and this affects the quality of their life. Public stigma that is also known as social stigma happens when the society in which a person lives endorses mental illness stereotypes and acts upon them in a discriminating way (Corrigan & Nieweglowski 2019). Self-stigma takes place when those who are stigmatised discriminate against themselves because of internalised prejudice, that is, they accommodate and perpetuate societal stereotypes towards themselves (Corrigan & Nieweglowski 2019). This is demonstrated by a study conducted by Yin et al. (2020) in which caregivers of people diagnosed with mental illness were showing a tendency of avoiding to be identified with a family member who has mental illness or to attend social events because of shame and embarrassment. However, families in different contexts of a country may have different experiences of courtesy stigma. This is largely because communities hold different stereotypes about mental illness and stigmatise mental illness in their sociocultural contexts (Becker et al. 2019; Makanjuola et al. 2016). Even though there is evidence from studies conducted in Botswana showing that families of people diagnosed with mental illness are stigmatised, there is paucity of published studies describing the experiences of courtesy stigma experienced by families of people diagnosed with mental illness in Lobatse (Modie-Moroka 2016). In addition, anecdotal evidence from a psychiatric hospital in Lobatse suggests that courtesy stigma of mental illness is a concern, with reports of diminished support by some families towards their member diagnosed with mental illness. This study, therefore, sought to explore and describe the experiences by families of people diagnosed with a mental illness as a result of courtesy stigma at the national psychiatric referral hospital in Lobatse, Botswana.

### Problem statement

Stigma towards people with mental illness and their families remains a problem despite advances in treatment and care approaches (Mascayano, Armijo & Yang 2015; McGinty et al. 2015). Families of people with mental illness suffer alienation, rejection and prejudice because of cultural or societal stereotypes of perceptions that these families are cursed or are punished for some wrongdoings (Chilale et al. 2017). Nevertheless, the family institution remains the mainstay and centre of strength for people with mental illness (Ebrahim et al. 2020). Empirical evidence shows that family members of people suffering a mental illness often experience stigma related to their relative’s mental illness (Van Der Sanden et al. 2015). This is confirmed by the results of a study by Reupert et al. (2021) in which relatives of people with mental illness were reported to be experiencing shame, disappointment, engendering hurt and being avoided by others. Moreover, Yin et al. (2020) posits that in the plight of being ostracised, the relatives of people diagnosed with mental illness employ selective disclosure as means learnt from their own experience and sociocultural context to cope with stigma. Some families resorted to prayer and religious support to cope with stigma of mental illness while others sought professional and social support (Iseselo, Kajula & Yahya-Malima 2016; Meshkinyazd, Bordbar & Heydari 2021). Regardless of the differences in socio-cultural influences, the stigma of mental illness has a negative bearing on the quality of life for people diagnosed with mental illness and their families. Although there is a dearth of studies conducted to establish the level of impact of the courtesy stigma of mental illness in Botswana, there is empirical evidence that communities in low-middle income countries such as Botswana are affected by stigma because of their complex community and cultural factors (Mascayano et al. 2015). In addition, the principal researcher’s experience as a nurse educator with constant contact with families of persons diagnosed with mental illness at a psychiatric hospital in Lobatse has encountered reports of patients lacking family support attributable to courtesy stigma of mental illness. Thus, this study explores experiences of families on the stigma of mental illness in Lobatse to close the literature gap and form a baseline for researchers to recommend guidelines for averting stigma of mental illness.

### Research question

- *What are the experiences of courtesy stigma of families with a member diagnosed with a mental illness in Lobatse, Botswana?*

### Research aim

- To explore and describe the experiences of courtesy stigma of families with a member diagnosed with a mental illness in Lobatse, Botswana.

## Methodology

### Study design

Qualitative contextual phenomenological design was used for the study and it enabled the researchers to explore the lived experiences of individual family members with a kin member diagnosed with a mental illness and describe them within the context in which they were experienced (Gray 2017). The design was suitable for this study as it sought to make meaning of the life-world of the participants by exploring and describing how they experienced courtesy stigma of mental illness and its influences on the quality of their lives.

### Study context

The study was conducted at an outpatient department (OPD) of a psychiatric hospital in Lobatse, one of the towns in the southern region of Botswana. The town is located in the South East District about 70 km from the capital city, Gaborone and has a population of about 29 772 people as per the year 2022 population and housing census (Statistics Botswana 2022). Lobatse gained its popularity around mental health services due to its rich history in the provision of specialised psychiatric care, which was first introduced by Dr Sbrana in this town. Currently, the selected psychiatric hospital is the only national referral psychiatric facility in the country with a bed capacity of about 300 patients (Otlhapile, Gitau & Kuria 2023).

### Population

The study population comprised family members with a person diagnosed with a mental illness who met the eligibility criteria and had accompanied a family member for psychiatric mental health services at an OPD of a psychiatric hospital in Lobatse, Botswana. About 600 – 700 patients with mental illness are followed up at OPD of this psychiatric hospital in a month and most of them under the company of a family member. Although there is evidence from literature that families of people diagnosed with mental illness face stigma because of their association with their mentally ill family member, there is limited research conducted in Lobatse, Botswana on courtesy stigma of mental illness (Shi et al. 2019).

### Sampling technique

The study used purposive sampling technique. This sampling method gave the researchers an opportunity to select study participants based on their judgement about the participants’ usefulness or representativeness for the study (Polit & Beck 2017). The sample was homogenously selected from consenting participants who shared a set of characteristics required for the study and who were accompanying a family member for follow-up care or review at OPD (see inclusion and exclusion criteria section for details). Only the individual family members who accompanied their kin diagnosed with a mental illness provided consent and answered the interview questions based on their own experiences. Purposive sampling afforded the researcher an opportunity to develop insight into the participants’ lived experiences of courtesy stigma and to describe their experiences within their context.

### Sampling size

Sample size refers to the number of participants that the researcher includes in the study (Polit & Beck 2017). The sample size for this study depended on data saturation when there were no new themes emerging in the data and redundancy had been realised (Gray 2023; Polit & Beck 2017). Of a sample size of 15 participants who were interviewed, data saturation was reached after 9 interviews. This is in line with suggestions of literature that the sample size that is between 5 and 50 participants is adequate for a qualitative study (Dworkin 2012).

### Inclusion criteria

To be eligible for this study, participants had to be:

- Persons who were currently staying with and taking care of someone with mental illness and related to him or her or being a guardian.
- Accompanying a person diagnosed with a mental illness to an OPD of a psychiatric hospital in the South East district in Botswana for mental health services.
- The only family member of a person diagnosed with a mental illness to participate in the study.
- Eighteen years of age and above and all genders.
- Staying with a person with mental illness in their homes for over 6 months.
- Prepared to be audio recorded.
- Willing to communicate in Setswana or English.

### Exclusion criteria

To be ineligible for this study, one must have been:

- Diagnosed with a mental illness.
- A carer who did not stay with the mentally ill family member but cared for him or her temporarily for example, those who reported to be staying with the patient only on weekends.
- Under the age of 18 years.
- A carer for the mentally ill person for less than 6 months.

### Pilot study

The researchers conducted pilot interviews with the first two participants to establish if the interview question was clear and focused on the study. The interviews were conducted telephonically and the calls were recorded with an audio call recorder with the permission of the participants. The two pilot interviews were included in the rest of the data set. Interviews conducted telephonically are a convenient way of collecting data, which does not require the researcher to travel to a specific place for the interviews and also spares the interviewees the hassles of travelling to meet interviewers (Hofisi, Hofisi & Mago 2014). As further opined by Hofisi et al. (2014), there is ‘facial anonymity’ in telephonic interviewing that may make the participants to open up and raise interesting and unanticipated issues in ways they would not in a face-to-face interview.

### Data collection

Data were collected from participants using in-depth individual interviews. The researchers developed an English interview guide, which was then translated to Setswana. The in-depth interviews were all conducted in Setswana, which was the preferred language for the participants. The interviews were conducted telephonically and they took about 40 min to 1 h for each participant, which is of no significant time difference with face-to-face interviews. As postulated by Johnson, Scheitle and Ecklund (2021), the main reason on slightly less duration appeared to lie in the intrinsic propensity for telephone interviewees to provide relatively less elaboration or detail. That still being the case, there seems to be a consensus in the literature that face-to-face interviews are the best format (the gold standard) compared with remote means. However, literature stipulates that in-depth interviews can still be conducted through remote methods such as the use of telephone because of logistical, practical, and safety reasons and be as effective as face-to-face interviews (Azad et al. 2021; Morris 2015). Moreover, there is a growing body of literature that supports the use of in-depth telephonic interviews as a viable and equivalent of face-to-face in-depth interviews, with some even arguing that telephonic interviews, in some regards, are methodologically superior to face-to-face interviews (Azad et al. 2021). Thus, telephonic in-depth interviews generate the same amount of data richness as face-to-face interviews in terms of topic-related information, even though telephonic interviews tend to be shorter (Azad et al. 2021). However, in order to enhance data quality that would have been compromised by the absence of non-verbal cues and difficulties in identifying visual emotional expressions in the telephonic interviews, the researchers intensified verbal feedback and follow-up probes, as well as using vocalisations and clarification for participants to show responsiveness (Azad et al. 2021). The move promoted careful listening and engagement by the participants throughout the interviews. To this end, the study utilised semi-structured interviews by means of an interview guide with a preconceived question that was appropriate in generating data to answer the research question and focus on the phenomenon under study. The researchers employed interpersonal communication techniques drawn from the field of psychotherapy and counselling such as probing, reflecting, exploring and seeking clarification (Gray 2023; Halter 2018). The researchers asked an open-ended phenomenological question followed by probes and ‘follow-ups’ for depth of information. The question asked was:

- *What are your experiences of stigma as a result of caring for a family member diagnosed with a mental illness?*

#### Data analysis

The qualitative data that were collected through an audio recorder in Setswana was transcribed verbatim, translated to English, and then independently analysed by both the researcher and the co-coder in harmony with the method of thematic analysis, that is, the six-phase approach of thematic data analysis (Polit & Beck 2017). The researcher sent raw data and work protocol to the co-coder for the duo to independently analyse the data. Then a meeting was held by the researcher and co-coder and consensus was reached on the themes of categories for the codes. Themes were categorised using abbreviations and symbols for the classification of words in the data. Data analysis ran concurrently with data collection.

### Approach to the six phases of thematic analysis

#### Familiarising self with data

According to Braun and Clarke (2012), this is the phase in which the researcher immerses self in the data set by reading and re-reading it and thus getting actively engaged with the searching for patterns of meaning. Thus, the researcher achieved familiarisation with data through reading and re-reading of the textual interview transcripts and listening to the audio recorded data.

#### Generating initial codes

During this phase, the researcher began to systematically analyse data through the use of codes (Braun & Clarke 2012). It is at this stage that the entire data set was organised into meaningful groups. First of all, data extracts were coded and then all data extracts bearing the same code were assembled (Herzog, Handke & Hitters 2019). The researcher assigned labels to the data that were potentially of relevance to the research objective.

#### Searching for themes

At this stage, analysis started to develop and the researcher’s focus shifted from codes to themes (Braun & Clarke 2012). Searching for themes was achieved through clustering together data that shared unifying attributes, reflecting an emerging coherent and meaningful pattern. The researcher then explored the relationship between the identified themes and combined them into overarching themes that built up a story from the data.

#### Reviewing potential themes

This stage involves a recursive process in which the emerging themes are reviewed against the coded data and the data set in its entirety (Braun & Clarke 2012). The researcher achieved this by exploring how the themes related to the data and in the process verified and maintained some themes, discarded those that did not address the research objective and relocated some themes under others that carried the same meaning.

#### Defining and naming themes

It is at this stage that the researcher reviewed the names of themes that specified the essence of each theme. The researcher explored the relevance of themes to the research objective and how the themes were relevant to the story the researcher wanted to tell about the data (Herzog et al. 2019). The themes were finalised after the renaming had led to satisfactory results (Table 2).

#### Producing the report

The whole exercise of producing the report involved choosing, ordering and chronicling the findings to produce an account of the data (Vaismoradi et al. 2016). It is at this stage that the researcher transformed the analysed data into an interpretable piece of writing through use of vivid examples that relate to the themes, the research question, and existing literature. Thus, the final report contained interview quotes from data extract, which best represented particular themes that emerged from the analysis.

### Trustworthiness

The researcher ensured the rigour of data collection and analysis by using the criteria according to Lincoln and Guba’s framework, that is, through credibility and dependability of the study, its transferability, and conformability (Polit & Beck 2017).

#### Credibility

As a criterion for evaluating data quality, credibility entails confidence on the truth value of data, which is obtained from the lived human experiences as experienced and perceived by informants (Polit & Beck 2017). Truth value is concerned with establishing confidence in the truth of the findings through accurate interpretation and description of the participants’ experiences that can immediately be recognised by people who also share the same experience (Polit & Beck 2017; Renjith et al. 2021). Thus, during the interviews, the researcher verified and validated information provided by the participants through follow-up questions for accurate interpretations. The credibility of this study was also enhanced through repeated listening to the interview audios for accurate interpretation and description of the families’ experiences of courtesy stigma. Then the researcher read and re-read transcripts for immersion into the data while compiling the final report and further used the direct words of the participants as strategies to strengthen the credibility of the study.

#### Dependability

It refers to the stability of data over time and conditions (Polit & Beck 2017). Literature points out that dependability can be achieved through strategies such as triangulation, use of an audit trail, stepwise replication, among others (Forero et al. 2018). The researcher ensured dependability of this study through the dense description of the study methods such as exact methods of data collection, analysis, and interpretation. To this end, the researcher kept an audit trail that accounted for all the research activities and decisions, that is, how the data were collected, recorded and analysed for other researchers to clearly follow. Accordingly, peer-examination by the supervisors who are experts in qualitative research enhanced dependability of this study.

#### Confirmability

It is the extent to which the study results can be certified by other researchers (Grove & Gray 2023; Polit & Beck 2017). Confirmability provides an assurance that the data represent the information as given by the participants to avert figments of the researcher’s imagination (Renjith et al. 2021). Thus, this study used the audit strategy as the major technique to establish confirmability (Grove & Gray 2023). To establish an audit trail, the researcher collected all the documentation on the study such as the interview scripts, field notes as evidence of the methodological processes that the researcher followed.

#### Transferability

This is the degree to which the findings of a study can be extrapolated to other settings (Polit & Beck 2017). Therefore, the researcher provided a detailed description of the research methods such as the recruitment inclusion criteria, the sampling techniques employed, and the data collection methods so that the study can be extrapolated to other contexts or settings.

### Ethical considerations

The Institutional Review Board at North-West University approved the research and provided an ethical approval for the study to be conducted. Permission to conduct the study was first solicited from the Ministry of Health and Wellness IRB (Ref: HPDME 13/18/1). Permission was also sought from Sbrana Psychiatric Hospital in Lobatse to conduct the data collection at their facility (Ref: SPH 4/2/12 I). Data were collected after the participants provided informed written consent. For anonymity, no identifying information was used and the data file was password-protected, with access limited to the lead investigator. Moreover, participants were assured of their right to self-determination, that is, they were allowed to choose to abstain or participate, their right to confidentiality and anonymity, justice, and protection from harm. Data such as recorded audio calls and field notes were erased immediately after being transferred to a password-protected computer for confidentiality. The co-coder also signed a confidentiality agreement as commitment to ensure confidentiality. The ethical principles of justice, beneficence and respect for human dignity were observed throughout the duration of the study.

## Results

### Demographic description

A total of 15 family members (*N* = 15) took part in the study and four of them (*n* = 4) were males while the rest were females constituting the highest number of the sample (*n* = 11) (Table 1). The participants were of a black race with ages ranging from 33 to 57 years, with mean age of 46 years (s.d. = 8.82). Of the 15 participants (*N* = 100%), two had primary education (13.3%), four had tertiary education (26.7%) while those with secondary education constituted the highest proportion of 60% (*n* = 9). The minimum period that their mentally ill member lived with a mental illness was a year, while the maximum was 47 years.

**TABLE 1:** Demographic profile of participants and their patients.

Participant	Gender	Age	Race	Level of education	Relationship with the patient	Patient diagnosis	Years staying with patient
1	F	40	Black	Secondary	Sister	Schizophrenia	16
2	F	51	Black	Tertiary	Daughter	Schizophrenia	12
3	M	52	Black	Secondary	Brother	Psychosis	24
4	F	53	Black	Secondary	Daughter	Schizophrenia	9
5	F	35	Black	Tertiary	Daughter	Schizophrenia	15
6	M	33	Black	Secondary	Brother	Psychosis	6
7	F	62	Black	Primary	Sister	Schizo-affective	14
8	F	33	Black	Tertiary	Cousin	Psychosis	3
9	F	50	Black	Primary	Mother	Schizo-affective	8
10	F	44	Black	Tertiary	Mother	Psychosis	1
11	F	40	Black	Secondary	Sister-in-law	Schizophrenia	11
12	F	50	Black	Secondary	Nephew	Schizophrenia	25
13	M	41	Black	Secondary	Son	Schizophrenia	7
14	M	49	Black	Secondary	Uncle	Schizophrenia	8
15	F	57	Black	Secondary	Son	Schizophrenia	21

**TABLE 2:** Themes and sub-themes on the family members’ experiences of courtesy stigma associated with mental illness.

Themes	Sub-themes
Family members’ experiences of received stigma	Rejection by relatives and friends Rejection by neighbours and the community Fear that mental illness is contagious Labelling and stereotyping by the community Rejection by employers and customers
Family members’ experiences of stigma by association	Family members blamed and shamed for the illness and deviant behaviours of their mentally ill patient Families are blamed for witchcraft and inheriting mental illness
Family members’ experiences of internal stigma	Negative emotions Compromised physical health

### Theme 1: Family members’ experiences of received stigma

The participants expressed experiences of stigmatisation in various forms such as received stigma. Received stigma was explained by the participants as experiences of rejection by family and friends, rejection by neighbours and the community, fear that mental illness is contagious, labelling by the community members and rejection by employers and customers as discussed in the following sub-headings.

#### Sub-theme 1: Rejection by relatives and friends

As a result of stigma of mental illness, some participants reported that they experienced broken or changed relationships with their relatives and friends. Participants expected their friends and kinship relationships to accept them but surprisingly, some relatives and friends also joined in stigmatising them. This stigma was experienced by participants as the lack of visits and rejection by friends and some family members when compared with when they did not have a family member with a mental illness. So, the received stigma led to diminished social networks as these families were alienated and rejected by their friends and relatives as a result of stigma of mental illness as shown in the quotes below:

‘You end up feeling demoralised because of lack of support or even visits by relatives and friends.’ (Participant 5, 35 years old, female)

‘Even a visit from friends … some people pull back … people you were used to as a family.’ (Participant 8, 33 years old, female)

In this study, participants provided their experiences of the stigmatising labels endured by their families who experienced stigmatisation. Compared with studies of Iseselo et al. (2016) and Meshkinyazd et al. (2021), the participants reported heightened received stigma experienced through the lack of support and courtesy visits by close relatives and friends, which was viewed as a sign of being ostracised. The findings of this study show that the friends who used to provide support to families with a member diagnosed with a mental illness also pulled back their support as indicated in the given quotes. Similarly, a study conducted by Abojabel and Werner (2019) corroborates the rejection that families with a member diagnosed with a mental illness go through by revealing sentiments by a participant who indicated that the friends they used to depend on turned against them. Furthermore, authors have also documented that the families with a member diagnosed with a mental illness experience received stigma through social distance, avoidance, abandonment and rejection by relatives and friends because of courtesy stigma of mental illness (Abojabel & Werner 2019; Reupert et al. 2021). Thus, as revealed by literature, these families became alienated and rejected and this made them to also withdraw from acquaintances, and avoid some social engagements in order to avoid being confronted with stigmatising reactions by others (Van der Sanden 2015).

#### Sub-theme 2: Rejection by neighbours and the community

Participants in this study reported that they experienced received stigma and rejection mainly from their neighbours. In spite of knowledge of the dire situation that families of people diagnosed with mental illness face, their neighbours were reported to stigmatise them more as experienced through exclusion in some social activities because their family was viewed as useless. These families also reported that they felt rejected and unwelcome in social events when people distanced themselves from them especially when they were in the company of their family member with a mental illness. As for the neighbours, the participants expected them to better understand their ordeals and support them as they are the daily witnesses of what they go through. Even though the community was reported to stigmatise these families, their neighbours were reported to demonstrate the most stigmatising tendencies such as being unwelcoming, shunning and laughing at them as shown in the following quotes:

‘Example is when I was excluded in some work to do in a social event and someone said, “will she manage this one?”’ (Participant 1, 40 years old, female)

‘I sometimes feel like fleeing given the way the neighbours shun and laugh at me.’ (Participant 6, 33 years old, male)

As part of experiences of received stigma, the families of people diagnosed with mental illness in this study experienced rejection and dehumanising labels from their neighbours and the community. In a study conducted by Hanafiah and Bortel (2015), participants confirmed that they face stigma of mental illness and that their communities stigmatise them because they hold oversimplified and negative images of mental illnesses, people diagnosed with mental illness and their families. In this study, the participants experienced exclusion in some social activities based on stereotypes held by their community that view them as incompetent. This finding is consistent with those of a study conducted by Van der Sanden et al. (2015) in which families with a member diagnosed with mental illness were labelled as incompetent and socially excluded. Furthermore, and consistent with the results of this study, the aforementioned author reported that families with a member diagnosed with mental illness also experienced rejection in the form of social distancing in which people did not want to associate with them. Therefore, the findings by Hanafiah and Bortel (2015) and Van der Sanden et al. (2015) are consistent with those of this study where community members displayed rejection and discriminatory behaviours such as showing unwelcoming attitude, shunning, among others, towards families of people diagnosed with mental illness and neighbours being more implicated.

#### Sub-theme 3: Fear that mental illness is contagious

Participants provided rich descriptions of their experiences of the stigmatising labels that they endured through their mental illness and are further extended to them because of their relationship with them. It was apparent in this study that the use of derogatory words by the perpetrators of stigma while referring to carers of people diagnosed with mental illness insinuated that they also had mental illness and that they contracted the illness from their family members who are mentally ill, thus painting a picture of mental illness as ‘contagious’ as indicated in the following quote:

‘It’s like mental illness is contagious.’ (Participant 8, 33 years old, female)

Furthermore, the negative comments towards families with a member diagnosed with mental illness in this study involved treatment of these families as if mental illness was contagious. This assertion was echoed by one of the participants who reported to have been viewed as though staying with the mentally ill translates to being like her patient. Although in this study the families of people diagnosed with mental illness were subsumed to have mental illness like their mentally ill members, the participant viewed this assertion as just reckless and for the purpose of stigmatising them with no overt fears that the disease is actually contagious. Comparably, a study conducted in Namibia revealed that mental illness was viewed as being actually contagious (Bartholomew 2018).

#### Sub-theme 4: Labelling and stereotyping by the community

The participants reported labelling as an experience that involved use of demeaning and negative remarks by perpetrators of stigma leading to feelings of provocation and hurt. This study reveals that the families with a member diagnosed with a mental illness were given labels by the community together with their mentally ill family members by the use of demeaning words such as ‘mad’, ‘insane’, ‘idiots’ towards them. The participants viewed the use of such labels and name calling towards them and their mentally ill as demeaning, dehumanising, shaming and making them feel embarrassed. Labelling and stereotyping as a form of received stigma included the use of impolite words towards families of people diagnosed with mental illness and other interpersonal stigmatising messages as indicated in the quotes that follow:

‘They use shaming and demeaning words such as, her mother is mad [*setseno*].’ (Participant 2, 51 years old, female)

‘As for some people, you cannot stand their remarks … they just speak anyhow and with no consideration of your feelings … with words like *dieleele* [fools], *dimatla* [the stupid].’ (Participant 6, 33 years old, male)

The findings of this study are in agreement with existing literature that suggests that families with a member diagnosed with mental illness experience discriminatory labelling utterances and stereotypes from the public in the form of demeaning names, restrictiveness and prejudice (Becker et al. 2019; Poreddi, Thimmaiah & Math 2015a). There is evidence from other studies that labelling and stereotyping of families of people diagnosed with mental illness by the community are common and include the use of verbal abusive and impolite words such as ‘mad’, towards these families. The results of this study also reveal that, families with a member diagnosed with a mental illness were labelled as ‘*ditseno*’, ‘*dieleele or diso*’ and ‘*dimatla*’ in local lingo, which roughly translates ‘the mad ones’, ‘fools’ and ‘the stupid ones’, respectively, used to justify discriminating them. Similarly, in another study also conducted in Botswana by Becker et al. (2019), the colloquial term, ‘*setseno*’ meaning ‘the person who is mad’ was used to label the mentally ill for their deviation from the social norms. The mentally ill receive the label ‘*setseno*’, which the perpetrators of stigma in this study pluralised to ‘*ditseno*’, a slang Setswana term for ‘the mad ones’ to extend the label to the families of people diagnosed with mental illness because of their relationship with their mentally ill family member. Such labelling and stereotyping is a worldwide phenomenon and has persisted from medieval times to date; for example, in Namibia, they label the mentally ill as ‘*omunanamwengu*’ which also translates to ‘mad one’ (Bartholomew 2018).

#### Sub-theme 5: Rejection by employers and customers

Received stigma from the public was reported to be having a tremendous impact on the lives of families of people diagnosed with mental illness with reports of annulled opportunities for employment and some disadvantaged in business prospects. The participants reported that their mentally ill family members are usually unkempt and that they are thus judged and rejected based on how their mentally ill family members appear. In the process, people also loathe the food they sell as a way of rejecting them. Furthermore, they reported a form of rejection in which they were denied employment opportunities because of their relationship with the mentally ill family member. Following are quotations that attest to the experiences of rejection that the families of people diagnosed with mental illness face:

‘Yes, people judge us based on our patient’s mental state and appearance and as I sell food, they loath what I sell too based on my patient’s appearance and this makes me to lose business opportunities.’ (Participant 5, 35 years old, female)

‘They tell me that they cannot hire caregivers of patients diagnosed with mental illness.’ (Participant 7, 62 years old, female)

The labels and stereotypes towards mental illness negate the economic opportunities for families of people diagnosed with mental illness. A study by Van der Sanden (2015) reported on missed career opportunities for caregivers of people diagnosed with mental illness and that it contributed to financial hardships. This study also documents participants’ experiences of rejection in which their potential employers denied them an opportunity for employment because they cared for a mentally ill family member leading to financial hardships. Nevertheless, there is dearth of literature on the impact of courtesy stigma on employment opportunities for families of people diagnosed with mental illness. In addition, families with a member diagnosed with a mental illness in this study also suffered the blow of less income from their businesses owing to their rejection as customers viewed them in the light of their mentally ill family member and loathed what they sold leading to diminished quality of life. However, studies are equivocal on received stigma and its effect on businesses for families who have a member diagnosed with a mental illness.

### Theme 2: Family members’ experiences of stigma by association

It was evident in this study that stigma by association is one of the most psychosocial burden that the families or caretakers of people diagnosed with mental illness faced in their lives. This type of stigma was reported to be having some negative implications on the quality of life of the family members of people diagnosed with mental illness as they experienced high levels of shame and embarrassment.

#### Sub-theme 1: Family members blamed and shamed for the illness and deviant behaviours of their mentally ill patient

Families of people diagnosed with mental illness are blamed for everything that surrounds their patients and this was evident from the participants’ assertions that centred on being blamed and shamed for both the onset of their patients’ illness and the deviant behaviours that they displayed as demonstrated in the following quotes :

‘When the people see the patient being unkempt, they conclude that the family is not taking care of him.’ (Participant 3, 52 years old, male)

‘We cannot be blamed for our patients’ illness … these families cannot be blamed for their patients’ indulgence in substances.’ (Participant 3, 52 years old, male)

The participants reported to be also experiencing associated stigma in which the community recklessly generalised the negative attributes they thought the people with mental illnesses possessed to their family members thus stigmatising them. The results of this study confirm available literature that purports courtesy stigma to consist of stereotypes of blame and shaming (Meshkinyazd et al. 2021). In this study, families with a member diagnosed with mental illness also suffered blame that emanated from stereotypic thinking by the public such as blaming them for their patients’ indulgence in substance use and their unhygienic state. Subsequently, these stereotypes led to discrimination, which characterises associated stigma of mental illness. Similarly, a study conducted by Yin et al. (2020) reveals that families of people diagnosed with mental illness suffered stereotyping opinions and discriminatory behaviours and were often blamed for the onset of mental illness and relapses of a family member with mental illness, with the public expectation that these families should be ashamed, avoided or pitied. To this end, the findings of this study are consistent with literature that families with a member diagnosed with mental illness are blamed and shamed because of associated stigma, which may erode their morale as caregivers resulting in their withdrawal of care to their mentally ill member.

#### Sub-theme 2: Families are blamed for witchcraft and inheriting mental illness

Some participants reported to be blamed for witchcraft as the cause for the onset of their family members’ mental illness and were left to their care resulting in caregiver role strain. Furthermore, some participants reported that they were also judged as having inherited mental illness from their relatives and that this was used to scorn them whenever they tried to say or do something as indicted in the following quotes:

‘I am labelled as having “inherited” my mother’s madness [*botsenwa*].’ (Participant 5, 35 years old, female)

‘They leave everything on me because to them, I am the one who bewitched him.’ (Participant 7, 62 year old, female)

Moreover, some participants in this study were accused of the onset of their patients’ illnesses, which was believed to be because of their witchcraft. This finding is supported by those of another study that was conducted in Botswana to investigate local explanatory models of mental illness (Becker et al. 2019). The aforementioned study reports that the stigma that the participants faced emanated from local customary dogmas that associate causes of mental illness with traditional beliefs such as witchcraft, demon possession, among others. Similar findings were reported in South Africa where families of people diagnosed with mental illness were also blamed for neglecting traditional practices resulting in occurrence of mental illness of their family member (Nxumalo & Mchunu 2017). Although in this study genetic inheritance was also associated with the cause of mental illness, Makanjuola et al. (2016) generally conclude that sub-Saharan African cultures subscribe to a belief that mental illnesses have a supernatural cause. Conversely, in Europe, the stigma of mental illness is attributed to biogenic causes and personal traits, although there is also ascription of supernatural causes (Lersner et al. 2019). A study conducted in India also reveals that mental illness is believed to be because of God’s punishment and supernatural powers although other main causes are believed to be personal weaknesses and genetic inheritance (Poreddi, Blrudu & Math 2015b).

### Theme 3: Family members’ experiences of internal stigma

The findings of this study show that although the families of people diagnosed with mental illness experienced stigma from the public, they also internalised the received stigma and, in the process, stigmatised themselves too. According to Corrigan and Nieweglowski (2019), self-stigma takes place when those who are stigmatised also discriminate against themselves because of internalised prejudice by accommodating and perpetuating societal stereotypes towards themselves. In this study, internal stigma manifested in different ways, namely through negative emotions, and compromised physical health. Following is the discussion of the sub-themes that emerged from internal stigma.

#### Sub-theme 1: Negative emotions

Self-stigmatisation was reported to cause negative emotions and extensive emotional distress in the families of people diagnosed with mental illness. Participants reported different psychological challenges that they experienced such as sadness, frustration, anger and some reporting strong emotions marked by feelings of wanting to flee their homes because of self-stigma emanating from being stigmatised. The following quotes attest to the experiences of negative emotions by families with a member diagnosed with a mental illness:

‘It really makes me feel sad.’ (Participant 1, 40 years old, female)

‘I avoid social events at all cost to avoid discussing the patient and being reminded that we are failures … that I can’t manage this or that … it stresses me up.’ (Participant 8, 33 years old, female)

As a result of both the associated and received stigma, the participants internalised the sigma and, as a result, suffered internal stigma in which they experienced negative emotions and compromised physical health resulting from being stigmatised. It is evident through literature that families of people diagnosed with mental illness face rejection and respond with feelings of anger, shame and fear (Abojabel & Werner 2019). This study unearthed various emotional circumstances that these families undergo such as sadness and frustration as a result of internal stigma. These results are consistent with a body of research that also demonstrates that family stigma can cause shame, anger, psychological distress and hopelessness, which impinge negatively on the quality of life for family members of people with mental illness (Abojabel & Werner 2019; Meshkinyazd et al. 2021; Van der Sanden 2015). Although participants in this study seemed to have relatively the same emotional reactions to stigma, it was apparent that some were too distressed to a point of wanting to relocate from their communities with the hope of finding a better life elsewhere while others in internalising the stigma had self-blame and avoided social events.

#### Sub-theme 2: Compromised physical health

In this study, the participants were worried about hypertension as the physical problem that they experienced and they attributed it to stress engendered by courtesy stigma. They were aware that the continued stress and worries they experienced about the courtesy stigma had a negative bearing on their physical health as shown in the quotes that follow:

‘I then realised that I will die from high blood pressure and even leave my dependents.’ (Participant 1, 40 years old, female)

‘I now have hypertension because of the stress.’ (Participant 5, 35 years old, female)

Comparable to the findings of this study, literature also provides evidence that families of people diagnosed with mental illness develop somatic or physical complaints such as inertia, fatigue, insomnia and several physical pains and aches, documented as physical symptoms emanating from psychological distress related to courtesy stigma (Van der Sanden 2015). However, the complaint raised in this study as a result of stress was high blood pressure.

## Conclusion and recommendations

Experiences of courtesy stigma can be very stressful and devastating for family members of people diagnosed with mental illness. These experiences often generate responses that require the families to adjust to the stressful situations imposed by stigma within the family and different social expectations placed upon them by society in the face of the daunting courtesy stigma and burden of caring for their patients. With the insights gained from the findings of this study, programmes geared towards supporting families of people diagnosed with a mental illness can be developed to help them make healthy adjustments to their situations, for example, family education and counselling programmes to aid their coping. The study raises awareness on stigma of mental illness and can form a base for interventions to mitigate the effects of courtesy stigma of mental illness. Thus, community meetings can be held to discuss the experiences of these families in the bid to gain support from their friends, neighbourhood and the community. In terms of research, available literature on mental health in Botswana provides inadequate guidance to inform policy and practice (Opondo et al. 2020). Thus, the findings of this study close the knowledge gap that exists on the family experiences of courtesy stigma of mental illness in Lobatse, Botswana. Moreover, the study can be beneficial in stimulating further nursing research, which is necessary for continuing advancements to the nursing profession and critical in promoting optimal nursing care to families of people diagnosed with a mental illness. Given the findings of this study, the researchers recommend that there is need to develop anti-stigma policies and strengthen the available laws by prescribing the use of more neutral or the person-first language such as *mona le bolwetsi jwa tlhalaoganyo* [a person with a mental illness] and prohibit the use of derogatory labels such as *setseno* [the mad one].

## Limitations

The study used qualitative contextual phenomenological design and thus cannot be generalised to families with a member diagnosed with a mental illness in other parts of Botswana or the world over. Nevertheless, the challenge was mitigated by clear and rich description of the research process and achieved the desired resonance as the findings certainly resonate. Lastly, data collection was conducted telephonically and telephone interviews have shortcomings in that the researcher cannot capture non-verbal nuances indicating emotions such as discomfort, anger, amusement, among others, which can be picked during a face-to-face interview. To this end, the researcher could not observe the participants for non-verbal body language, which compromised the complete observation.
